# Supplementary material for: Frechet-power function distribution:Theory, properties and applications
Source: PLoS One. 2025 Dec 2;20(12):e0337823. doi: 10.1371/journal.pone.0337823 (PMC12671835; doi:10.1371/journal.pone.0337823)
Supplement: Attachment — Submitted filename: Review Report.docx [file pone.0337823.s001.docx]

**Review Report – Manuscript ID: PONE-D-25-39356**
**Title:** Fréchet–Power Function Distribution: Theory, Properties and Applications

**Recommendation:** Major Revision

### ****Summary****

This manuscript introduces the Fréchet–Power Function (FPF) distribution, a novel bounded-support model with heavy-tailed flexibility, designed to overcome limitations in existing bounded lifetime models. The authors provide theoretical development, statistical properties, estimation procedures, simulation studies, and real-world applications. The work is well-motivated and mathematically sound, and it addresses a genuine gap in the literature.

However, the manuscript would benefit from significant revisions to improve clarity, strengthen empirical validation, and better position the model within the broader statistical literature. My detailed comments are organized below.

**Abstract**

- **Strengths:** Clear statement of novelty; highlights applications in survival analysis and reliability engineering; mentions key statistical properties and estimation methods.
- **Concerns:**
  - Should briefly emphasize that the FPF distribution nests the Power Function distribution as a special case.
  - Could add a short sentence summarizing comparative results to immediately convey empirical strength.

**Suggestion:** Add “…including the Power Function distribution as a special case” and one quantitative phrase like “yielding lower AIC and BIC values than competing models in tested datasets.”

**Introduction**

- **Strengths:** Good coverage of bounded and heavy-tailed model families; well-motivated need for combining these traits.
- **Concerns:**
  - Literature review is long but lacks clear structural grouping (bounded models, heavy-tailed models, hybrids).
  - Multiple references to similar model families (Exponentiated PF, Beta-PF, Transmuted PF) could be synthesized in a comparative table.
  - Need more detail on why previous bounded models fail to capture extreme tails.

**Suggestion:** Add a table summarizing key models, their support, tail behavior, and hazard shape flexibility. Explicitly highlight that FPF fills a missing quadrant in this table.

**Section 2 – Proposed Distribution**

- **Strengths:** Clear mathematical formulation; derivations are logically sound.
- **Concerns:**
  - Inconsistent notation: switching between αθ and θα.
  - Repetition of substitutions in proof of Theorem 1 could be condensed.
  - Equation (3) has “generatoring” instead of “generating.”

**Suggestion:** Standardize notation to αθ throughout, streamline proof steps, and correct typographical errors.

**Section 3 – Survival and Hazard Functions**

- **Strengths:** Demonstrates flexibility in hazard shapes; ties parameter combinations to hazard behaviors.
- **Concerns:**
  - No discussion on parameter identifiability—whether multiple (α, θ) pairs yield indistinguishable hazard curves.
  - Figures 1–3 (descriptions given) need explicit legends, parameter labels, and axis descriptions for standalone interpretation.

**Suggestion:** Include a hazard-shape parameter map for α vs. θ showing regions of increasing, decreasing, and bathtub-shaped hazards.

**Section 4 – Graphical Illustration**

- **Strengths:** Qualitatively supports claims about flexibility.
- **Concerns:**
  - Missing numeric parameter annotations in the figures.
  - Could benefit from direct side-by-side plots with competing models for the same data.

**Suggestion:** Add comparative plots with PF, Kumaraswamy–PF, and Beta–PF under the same parameterization.

**Section 5 – Quantile Function**

- **Strengths:** Provides an explicit inverse CDF, facilitating simulation.
- **Concerns:**
  - No numerical illustration or example showing how quantiles vary with α and θ.

**Suggestion:** Include a table of selected quantiles for different parameter values to show practical differences.

**Section 6 – Moments and Related Measures**

- **Strengths:** Comprehensive derivations of mean, r-th moments, skewness, kurtosis, and MGF.
- **Concerns:**
  - Some integrals are shown in long form with repeated substitutions—could be moved to an appendix.
  - Special cases (α=1, θ=1) are mentioned earlier but could be reiterated here for context.

**Suggestion:** Move lengthy derivations to supplementary materials, leaving main text focused on results and interpretations.

**Section 7 – Estimation**

- **Strengths:** MLE derivations are correctly set up.
- **Concerns:**
  - No discussion of numerical optimization settings (starting values, convergence criteria).
  - No coverage probability analysis for MLEs.

**Suggestion:** Report the numerical optimization method, convergence tolerances, and add a bootstrap study to check confidence interval accuracy.

**Section 8 – Simulation Study**

- **Strengths:** Uses multiple sample sizes and parameter combinations; bias and MSE trends are clearly shown.
- **Concerns:**
  - Only bias and MSE are reported—no confidence interval coverage or computational performance metrics.
  - Acceptance–Rejection method efficiency not quantified.

**Suggestion:** Include acceptance rates for AR sampling, and report coverage probabilities for α and θ estimates.

**Section 9 – Application**

- **Strengths:** Real-world demonstration increases relevance.
- **Concerns:**
  - Methodology for model comparison not fully detailed—criteria (AIC, BIC, K–S) should be explicitly stated.
  - No statistical significance testing of differences between models.

**Suggestion:** Use likelihood ratio tests or Vuong tests to statistically compare model fits.

**Additional General Comments**

- Ensure PLOS ONE reference style consistency.
- Proofread for typos (“generatoring”, “Lex X∼”).
- Include a brief limitations section discussing the assumption of known (a, b) and sensitivity to misspecification.
