# Supplementary material for: Frechet-power function distribution:Theory, properties and applications
Source: PLoS One. 2025 Dec 2;20(12):e0337823. doi: 10.1371/journal.pone.0337823 (PMC12671835; doi:10.1371/journal.pone.0337823)
Supplement: Attachment — Submitted filename: Response to Reviewers.docx [file pone.0337823.s003.docx]

**Dear Editor and Reviewers,**

We sincerely thank you for the careful review of our manuscript titled *“Fréchet–Power Function Distribution: Theory, Properties and Applications.”* We appreciate the constructive comments and suggestions, which have greatly helped us to improve the clarity, rigor, and impact of our work.

Below, we provide a detailed point-by-point response to each reviewer’s comments. All changes are highlighted in blue color in the revised manuscript.

**Reviewer #1**

1. *Positioning in Literature:*
   I thank the reviewer for this important suggestion. In the revised manuscript (see introduction section), we added a comparative table summarizing existing bounded, heavy-tailed, and hybrid models, highlighting how the proposed FPF distribution differs in terms of support, tail behavior, and hazard flexibility(see introduction section) .
2. *Methodological Clarity:*
   We appreciate the reviewer’s concern regarding Theorem 1. After careful revision, we have removed redundant steps and ensured consistent notation (αθ vs. θα). We believe that Theorem 1 is central to establishing the validity of the proposed FPF distribution, and thus it remains in the main text rather than being moved to the supplementary material.
3. *Parameter Identifiability:*
   We have added a hazard shapes map. Specifically, we show that different parameter combinations can produce distinct hazard behaviors (See Figure 1).
4. *Simulation Study:*
   The simulation study has been expanded to include coverage probabilities, bootstrap-based analysis, computational runtime, and acceptance–rejection efficiency.
5. *Applications and Model Comparison:*
   The application section now includes multiple criteria (AIC, BIC, KS, Anderson–Darling) and model comparison tests (likelihood ratio and Vuong tests), with detailed explanation of estimation methods used for competitor models.
6. *Additional application to a non-survival bounded dataset:*

We thank the reviewer for this insightful suggestion. In response, we have included an additional application of the FPF distribution to a non-survival bounded dataset

7. *Assumption of Known Bounds:*
   We acknowledge that the FPF distribution assumes known support boundaries (a,b). We have noted this as a limitation of the study in the **Limitations** section and suggest that future research could explore the impact of uncertainty in the bounds on parameter estimation and model performance.
8. *Minor Points:*
   All typographical errors and inconsistent notation were corrected. Figure captions now include parameter annotations. Editorial artifacts were removed. We note that the Power Function distribution is not a special case of the proposed Fréchet–Power Function (FPF) distribution, because the Fréchet generator introduces an additional transformation that cannot be reduced to the original Power Function form. Instead, the abstract has been revised to highlight the empirical advantages of the FPF distribution, including its flexibility in modeling bounded, skewed, and heavy-tailed data.
9. **Reviewer #2**
1. *Motivation in Introduction:*
   We revised the introduction to strengthen the justification for the new model. We now emphasize limitations of existing bounded and heavy-tailed models and explain why combining them addresses an important gap.
2. *Structure:*
   The manuscript has been reorganized with clearer section divisions, improving readability.
3. *Figures:*
   I appreciate reviewers. All plots are now included with revised captions (Sections X–Y).
4. *KS Test Issue:*
   We added a careful discussion clarifying that a non-significant KS test p-value does not indicate poor fit, but rather failure to reject the null hypothesis that the data follow the model. The discussion now explains that this is expected when the model fits well, and why complementary tests (AIC, BIC, AD) are also used.
5. *Recent References:*
   We updated the literature review to ensure up-to-date relevance.
6. *English Editing:*

We thank the reviewer for the comment. The manuscript has been carefully edited to improve grammar, clarity, and overall readability, ensuring that the presentation of ideas is clear and concise throughout the paper.

**Conclusion**
We hope the revised version addresses all concerns and improves the manuscript substantially. We thank the reviewers again for their insightful feedback.

**Sincerely,**

Merga Abdissa Aga

Author
