# Supplementary material for: Frechet-power function distribution:Theory, properties and applications
Source: PLoS One. 2025 Dec 2;20(12):e0337823. doi: 10.1371/journal.pone.0337823 (PMC12671835; doi:10.1371/journal.pone.0337823)
Supplement: Attachment — Submitted filename: Response to Reviewers Plos.docx [file pone.0337823.s004.docx]

**Responses to Reviewer**

We sincerely thank the reviewers and the editor for their constructive and thoughtful comments. The feedback has been invaluable in improving the clarity, quality, and overall presentation of our manuscript. We have carefully addressed all suggestions, and the manuscript has been revised accordingly**.**

**Response to Reviewer #1**

**Comment 1:**
Include at least one comparative plot overlaying FPF with 2–3 competing bounded models on the same empirical dataset.

**Response:** We sincerely thank the reviewer for this thoughtful suggestion. Comparative analyses with competing bounded models have already been provided using histogram overlays and several goodness-of-fit measures (AIC, BIC, KS, etc.). These adequately demonstrate the model’s superior fit; therefore, we respectfully retain the current presentation.

**Comment 2:**
Expand limitations to include identifiability, boundary misspecification, and small-sample behavior.

**Response:** We thank the reviewer for the valuable guidance. The limitation section has been revised accordingly to include these points.

**Response to Reviewer #3**

**Comment 1:**
Reference number 5 is incorrectly formatted.

**Response:**
Corrected as suggested, and all references were checked for consistency with the journal style.

**Comment 2:**
Correct grammatical error in Theorem 1.

**Response:** Revised as suggested.

**Comment 3:**
Include three additional relevant papers.

**Response:** The suggested papers have been added to the reference list.

**Comment 4:** Add a short introduction before survival and hazard functions.

**Response:** A brief introductory paragraph has been added.

**Comment 5:**
Correct citation of “McGilchrist C. and Aisbett C. [31]”.

**Response:** Corrected as suggested.

**Comment 6:**
Add discussion on limitations and future directions.

**Response:** The limitation section have been updated accordingly.
